# Identification of tidal trapping of microplastics in a temperate salt marsh system using sea surface microlayer sampling

**DOI:** 10.1038/s41598-020-70306-5

**Published:** 2020-08-24

**Authors:** Jessica L. Stead, Andrew B. Cundy, Malcolm D. Hudson, Charlie E. L. Thompson, Ian D. Williams, Andrea E. Russell, Katsiaryna Pabortsava

**Affiliations:** 1 University of Southampton, Southampton, SO14 3ZH UK; 2 Channel Coastal Observatory, Southampton, SO14 3ZH UK; 3 National Oceanography Centre, Southampton, SO14 3ZH UK

**Keywords:** Environmental impact, Ocean sciences

## Abstract

Microplastics are contaminants of increasing global environmental concern. Estuaries are a major transport pathway for land-derived plastics to the open ocean but are relatively understudied compared to coastal and open marine environments. The role of the “estuarine filter”, by which the supply of sediments and contaminants to the sea is moderated by processes including vegetative trapping and particle flocculation, remains poorly defined for microplastics land to sea transfer. Here, we focus on the sea surface microlayer (SML) as a vector for microplastics, and use SML sampling to assess microplastic trapping in a temperate marsh system in Southampton Water, UK. The SML is known to concentrate microplastics relative to the underlying water and is the first part of rising tidal waters to traverse intertidal and upper tidal surfaces. Sampling a salt marsh creek at high temporal resolution allowed assessment of microplastics in-wash and outflow from the salt marsh, and its relationship with tidal state and bulk suspended sediment concentrations (SSC), over spring and neap tides. A statistically significant decrease in microplastics abundance from the flood tide to the ebb tide was found, and a weak positive relationship with SSC observed.

## Introduction

Microplastics are defined as “any synthetic solid particles or polymeric matrix, with regular or irregular shape and size, and with size ranging from 1 µm to 5 mm, of either primary or secondary manufacturing origin, which are insoluble in water”^1^. They are of increasing environmental concern due to their ubiquitous presence in oceans^2^, rivers^3,4^, the atmosphere^5^ and on land^6^. Microplastics are also more abundant by quantity compared to larger meso- or macroplastic debris^2^. It is currently estimated that 80% of marine plastic debris is derived from land-based anthropogenic sources^7^, although these estimates are highly uncertain^7^. Between 1.15 and 2.47 million tonnes of plastic debris of any size larger than 300 µm is estimated to be transported by rivers^3^. Due to this large plastic throughflow, estuaries are recognised as an important transport pathway from land to sea for microplastics^8^. As well as these riverine inputs, estuaries are frequently sites of intense urbanisation and industrial development, and receive plastic inputs from these sources directly, including through discharges from storm drains and waste water treatment works^9^.

Estuaries are, however, relatively understudied compared to beach and open marine environments with respect to both macro- and micro-plastics^10,11^, despite their likely importance for microplastic land-sea transfer and their ecological importance. Estuarine habitats such as salt marshes and mudflats are also potentially more favourable for the deposition of microplastics over high-energy environments such as sandy beaches^12^. While several estuaries worldwide have been sampled to determine the abundance of microplastics (e.g.^13–15^), it is only recently that detailed studies of microplastics cycling and trapping in estuaries have begun to appear in the published literature (e.g.^16,17^).

Estuaries are widely recognised as having a filtering effect on sediment and for anthropogenic contaminants^9,18^. The estuarine filter comprises a number of mechanisms, including vegetative trapping and particle flocculation, which moderate the supply of sediment and contaminants to the sea. Estuarine systems including coastal wetlands (e.g. tidal and subtidal flats, salt marshes, lagoons and mangroves) act as sinks for sediments as well as for anthropogenic contaminants^19,20^, potentially including microplastics^17,21^.

This study assesses the potential for vegetative (and wider physical) trapping of microplastics within a temperate salt marsh system over the flood-ebb tidal cycle. Vegetative trapping of larger plastic debris (> 5 mm) is well-recorded^22,23^, and is one of the mechanisms by which sediment is deposited in salt marshes and similar coastal wetlands^24^. Trapping occurs through a variety of processes, including particle capture by stems and leaves, and settling following a reduction in flow velocity through plants^25^. As vegetation effectively traps sediment and macro debris, it has recently been suggested that wetland vegetation may be an effective trap for microplastics^26^.

**Figure 1:**
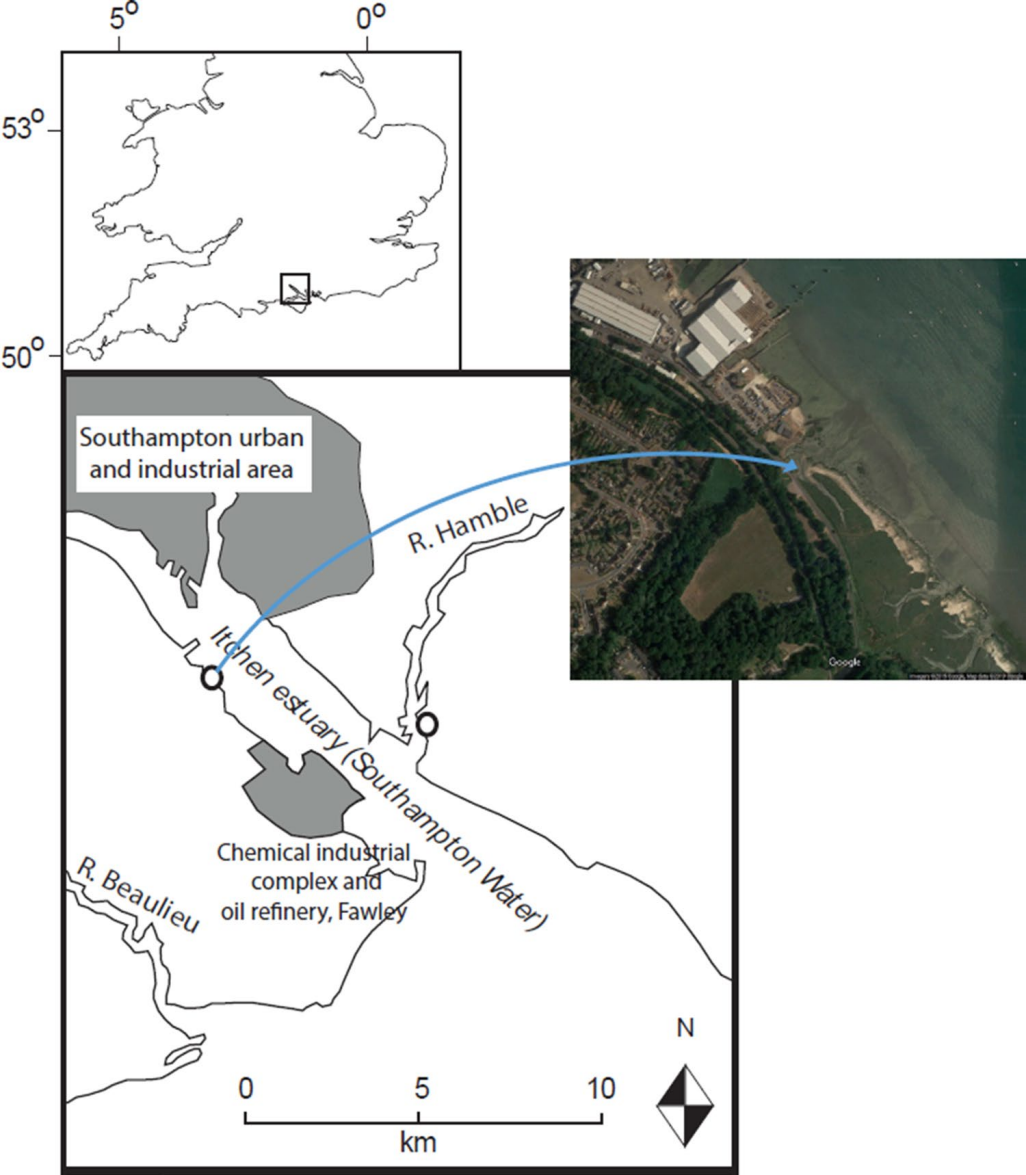
Location of the sampling site at Hythe, Southampton, UK. Aerial photographic imagery Copyright 2017 Google. Map data Copyright 2017 Google.

Here, we examine differences in microplastics abundance and characteristics in the sea surface microlayer (SML) during a tidal cycle at both spring and neap tides in a salt marsh creek in Hythe, Southampton Water, U.K. (a major urbanised and industrialised estuary in southern England, Fig. 1). Creek sampling over flood and ebb tides is used to provide an assessment of integrated microplastics inputs to and outputs from the marsh interior, via the SML. This provides the first (to our knowledge) detailed assessment of trapping processes of microplastics in a temperate salt marsh system based on tidal cycle sampling.

## Results

Suspected microplastic morphology was dominated by fibres, with over 700 fibres observed and three fragments. Microfibres were identified in 100% of SML samples taken over two sampling days. Median abundance over all samples was 7.5 fibres/m^2^. Minimum and maximum abundance on the spring tide was 1.9 fibres/m^2^ and 22.4 fibres/m^2^. On the neap tide, abundance ranged between 0.3 fibres/m^2^ and 7.1 fibres/m^2^. Both fibre abundance and suspended sediment concentration were non-normally distributed (Shapiro-Wilks test).

### Microplastics abundance on flood and ebb tides

**Figure 2:**
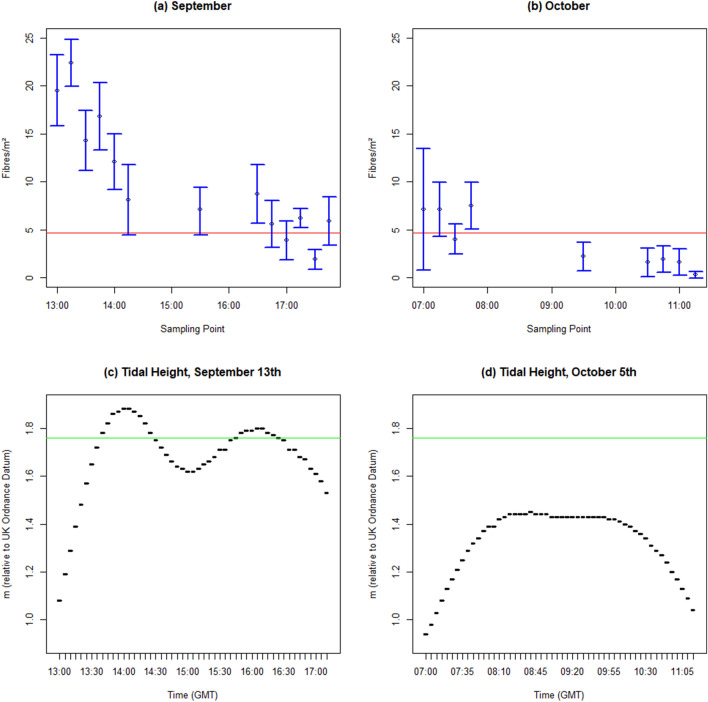
Fibre counts over a spring tide, 13th September 2018 (**a**), and a neap tide, 5th October 2018 (**b**); and tidal height curves for the sampling days, 13th September (**c**) and October 5th (**d**) (tidal height data measured at Dock Head, Southampton, and obtained from sotonmet.co.uk; horizontal green line indicates the marsh elevation).

Temporal trends in microfibre concentration over the flood and ebb tide are indicated in Fig. 2 for both sampling days, together with the calculated Limit of Detection (red line at y = 5). Both time series show a broad decline in SML microfibre concentration over the sampling period, with higher concentrations observed on the flood tide than on the ebb tide (although several points are close to or below the calculated limit of detection, particularly on the ebb tidal samples). Following a log-normal transformation of fibre abundance, a two-way ANOVA was carried out which showed a significant difference in fibre abundance between flood and ebb tides (*F* = 10.5553, *p* = 0.0011939), and between sampling days (*F* = 18.5052, *p* = 0.0005486). These differences were confirmed via Kruskal–Wallis tests on non-transformed data (for September, chi-squared = 7.4103, *p* = 0.006485, and for October, chi-squared = 6.7797, *p* = 0.03371).

When considering any relationship between suspended sediment concentration at 5 cm water depth (calculated from bulk water samples) and fibre abundance in the SML, a Spearman’s rank correlation (*ρ*) was used as neither variable was normally distributed (Shapiro’s Test: SSC *p* = 5.505e−06, fibre abundance *p* = 0.01319). This gave a result of a weak positive correlation (*ρ* = 0.5235, *p * = 0.01241) between the suspended sediment concentration and the fibre abundance.

### Microplastic characteristics

**Figure 3:**
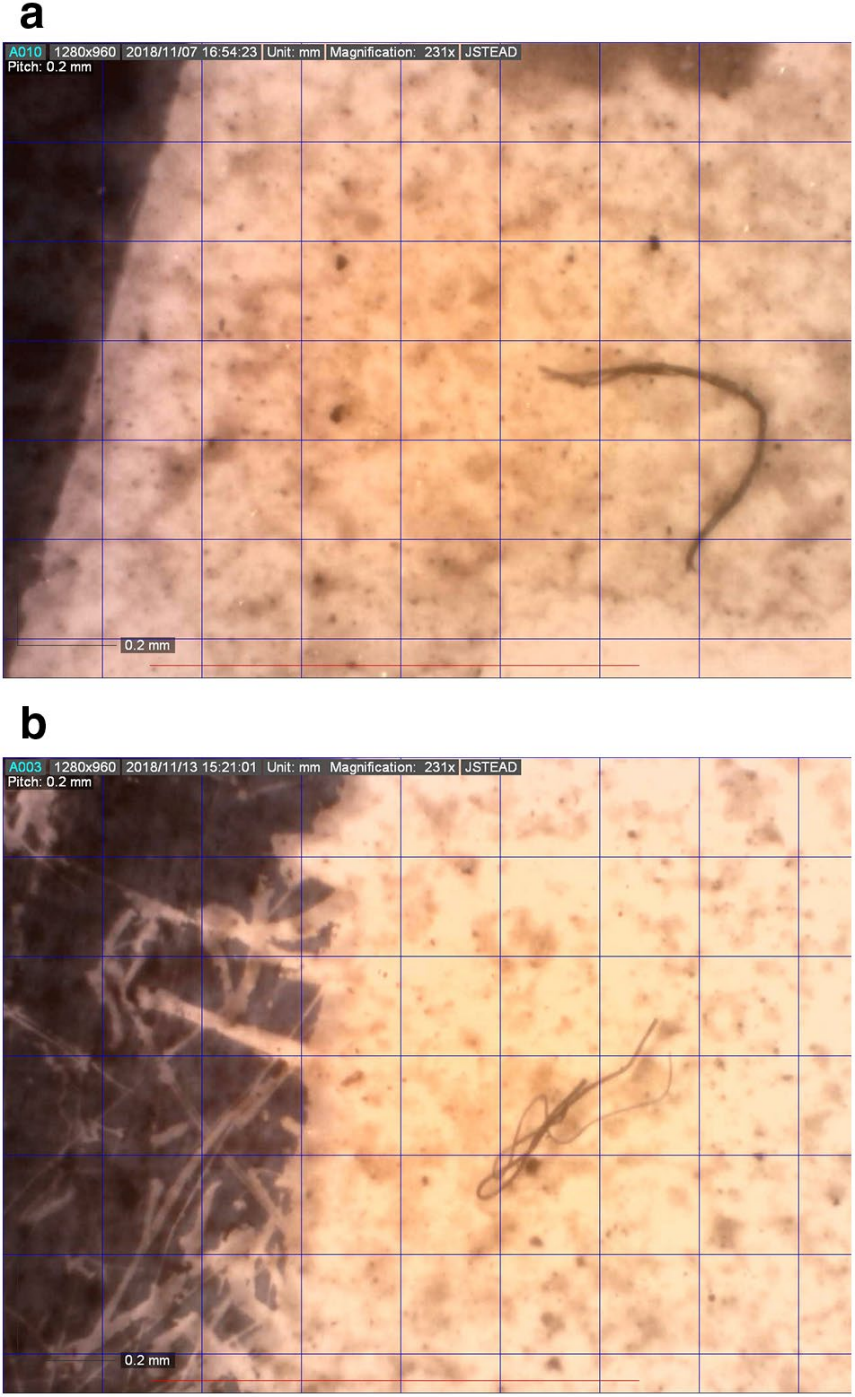
Images of fibres. (**a**) A frayed fibre. (**b**) A fibre tangled around itself. Scale: each square is 0.2 mm by 0.2 mm.

Standard light microscopy revealed that the fibres present were predominantly black in colour. 75% of fibres were black, 22% of fibres were blue and 2% were red. Some fibres displayed signs of weathering including fraying (Fig. 3a). Some fibres were found tangled (Fig. 3b).

Polymer identification was carried out using Fourier Transform Infrared Spectroscopy (FTIR). Of the 32 fibres tested, 21 returned spectrum matches of > 80%. 13 of these matched to polyethylene (62%), and one was identified as polyvinyl alcohol. One fibre was identified as cellulose nitrate (the material from which the initial filters were made), and was deemed to be a by-product of the re-filtration; and 6 were identified as cellulose or cellulose by-products (29%).

Previous studies on microplastics in Southampton Water found use of FTIR problematic in identifying polymer types, with no tested samples having a > 80% match to a spectrum in the polymer library^14^. This contrasts to the present study, however, it is likely that different methods were used to prepare samples for analysis. The three plastic polymers previously identified in Southampton Water were: cellophane, polyethylene and polypropylene^14^; one of which (polyethylene) was identified in the present study. Polyethylene is one of the most in-demand polymers^27^, utilised for packaging including plastic bags, as well as for ropes and fishing nets. These are all potential sources of the fibres recovered in this study.

**Figure 4:**
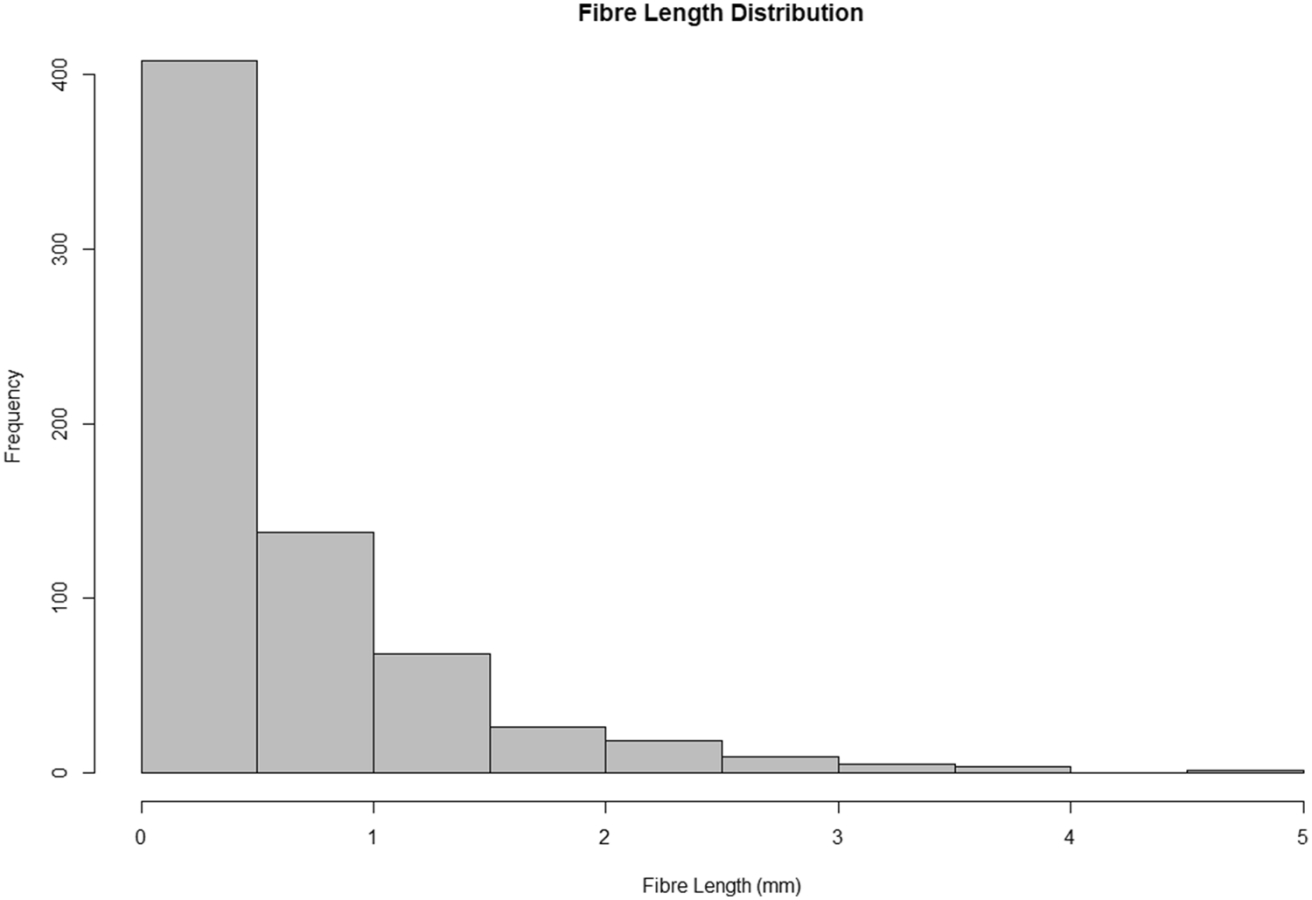
Fibre length distribution.

Several fibres with a length greater than 5 mm were found, which were outside our definition of microplastics (< 5 mm) and so were removed from the analysis. Most fibres (81%) were shorter than 1 mm in length. Fibre length distribution was found to be quasi-exponential, with significantly more smaller fibres than larger (Fig. 4). This follows previous studies which find that environmental microplastics are dominated by the smallest size class < 0.5 mm, which in this study made up 60.8% of the total counts^28–30^.

There was no significant difference between fibre lengths found in flood and ebb tide samples, as assessed by a one-way ANOVA. In order to assess for any differences in the proportion of shorter fibres, data were converted to percentage < 1 mm, as there were differing totals of fibres in each sample, and this passed a normality test (Shapiro–Wilk test, *p* = 0.19). There were no significant differences between fibre length for spring tide, neap tide and both sampling days combined.

### Counting error

**Figure 5:**
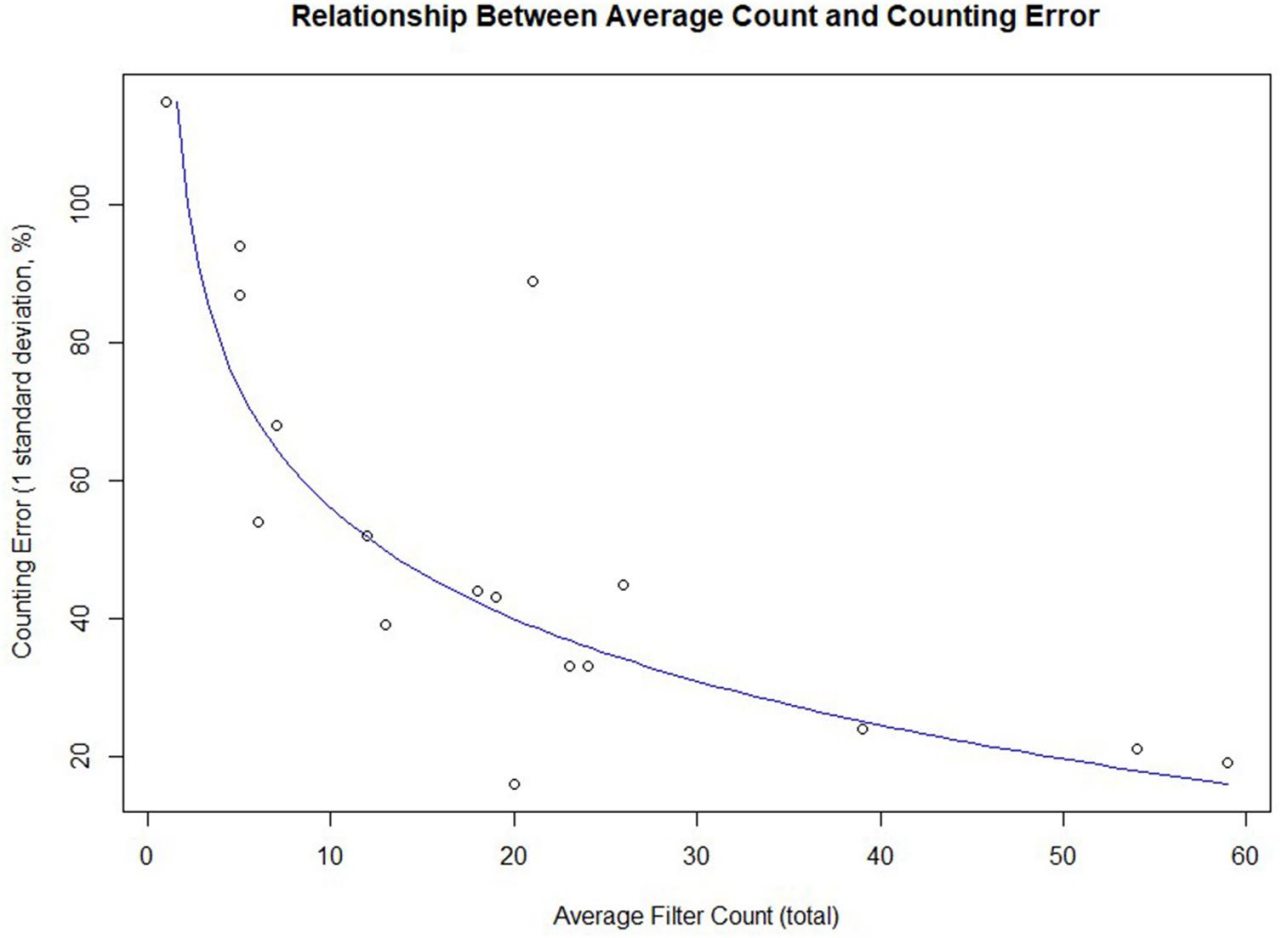
Relationship between average count and counting error.

Plotting average counts against counting error (Fig. 5) shows a relationship with an R^2^ value of 0.6881, and a counting error that approaches 10% at higher average count values. Counting error is frequently not assessed in the microplastics literature (unlike in other fields involving visual enumeration, such as pollen^31^ or micropalaeontological analysis^32^), but (as illustrated by Fig. 2) has significant implications for accurate visual enumeration of microplastics in less polluted environments.

## Discussion

The results presented here show that there is a comparable abundance of suspected microplastics in the SML in Southampton Water to that observed in previous work on adjacent estuaries^8^. The glass plate sampling method provides a rapid method for SML sampling for microplastics in estuaries^8^—here, we also assess and constrain counting uncertainties (based on different operators) and lower limits of detection (based on analytical blanks) to allow more robust comparison of data differences between samples.

The comparison of microfibre abundance between the flood tide entering the marsh creek and the ebb tide leaving the marsh creek (Fig. 2) supports the hypothesis that there is trapping of microfibres in the salt marsh system. There are significantly fewer microfibres in the ebb tide SML samples than the flood tide SML samples*,* and in October, the observed values on the ebb tide fell below the calculated limit of detection. There is a greater difference between flood and ebb tides in September (spring tide) sampling when the marsh floods to a greater depth than in October (tide height was a maximum of 1.88 m in September, 1.45 m in October). The bulk of the marsh vegetation at Hythe is not flooded on the neap tide due to the lower tidal height (Fig. 2c, d), leading to less vegetative trapping as the main marsh platform is not flooded. However, even without flooding the marsh in its entirety, there is still some sedimentation of microfibres during the long high water period, and possible trapping by direct interaction of MPs with exposed sediment and algal mats and other biofilms in the creek margins. Therefore, we propose that vegetative trapping is not the only process resulting in microfibres being retained in the upper intertidal in estuaries.

Previous studies investigating the deposition of microplastics in *Spartina sp.*-dominated marshes concluded that no or little trapping of microplastics occurs in these salt marshes^17,21^. However, the present study indicates a significant degree of trapping, with a *ca*. 2/3 decrease in microfibre abundance between the flood and ebb tides. The different conclusions between the present study and the literature could be attributed to the different methodologies used. In this study we did not investigate microplastic abundance in sediment or on vegetation, but rather sampled creek inflow and drainage over tidal cycles. Such an approach provides a more integrated measure of any trapping occurring, rather than a point sampling approach within the marsh. Another explanation is that the morphology of the Hythe marsh was more diverse in terms of plant species present compared to the two marshes sampled by Yao et al*.*^17^ and Cozzolino et al*.*^21^. It is clear that additional studies on a variety of salt marshes, through a range of methods and in different locations, are needed in order to confirm that there is a consistent trapping effect for microplastics by salt marshes, and the physical, chemical and biological controls on this.

The implication of the trapping of microplastics by salt marshes could be significant. While salt marshes have typically low biodiversity, they support considerable biomass^33^, and estuaries are very productive environments^34^. The presence and trapping of microplastics in a variety of environments within the upper intertidal zone (in the SML, and potentially on vegetation stems and leaves and in surface sediments) exposes a greater number of species with a variety of feeding modes to microplastics ingestion*.* Conversely, trapping and subsequent burial of microplastics in accumulating marsh sediments may effectively sequester microplastics and remove them from the water column and estuarine transport pathways, although further integrated data on estuarine fluxes are needed to assess the overall contribution of the upper intertidal zone to MP removal in relation to bulk cross-estuary transfer.

As observed for fibre abundance, a pattern of significantly lower concentration on the ebb tide compared to the flood tide is seen for the bulk suspended sediment concentration (SSC) at 5 cm water depth, and the difference is more significant for the spring tide sampled. When directly comparing the SSC and fibre abundance, a weak but significant positive correlation is seen (Spearman’s ρ = 0.433, *p* < 0.05). This supports the hypothesis of intertidal trapping for microplastics acting in a similar way to that for suspended sediments, but does not provide conclusive evidence that microplastics can be treated the same as sediment particles when utilising modelling to determine their fate or transport in estuaries.

Suspected microplastic morphology was, as in the previous study utilising the same sampling method^8^, dominated by fibres (> 99%). As such, only fibres are presented in the data here. As for the previous study^8^, it may be that the presence of sediment and organic material on the filters obscured microplastics of different shapes. The sampling technique may also have influenced the microplastics sampled, as previous studies in the open ocean found an effect of the SML sampling technique used on the abundance of various shapes of microplastic in the SML^29^. Numerous studies, however, have found a dominance of fibres in recovered plastics < 5 mm^35–39^.

The only previous study using a glass plate methodology to examine microplastic abundance in the sea surface microlayer is presented in Anderson et al.^8^. Average abundance in the Hamble estuary (draining into the east of Southampton Water) from that study was 6.93 fibres/m^2^. The current study showed an average abundance of 5.9 fibres/m^2^. This indicates a low but consistent presence of suspected microplastics in the wider Southampton Water system, with samples taken fifteen months apart.

Comparison to other studies is complicated by the use of alternate SML sampling methods. Studies sampling the SML for microplastics have variously used a rotating drum sampler^40^; a metal sieve^28,30^, or a stainless tray sampler^41^. These studies also utilise different units for the abundances found, but some comparison is possible. The abundance in the present study is higher than the abundances observed in two estuaries in the USA, Charleston Harbour and Winyah Bay^42^, which compare well morphologically to Southampton Water. The mean abundance in this study was 75.4 fibres/L, which is over double that observed in Winyah Bay (30.8 particles/L).

## Conclusions

An estuarine filter for microplastics was investigated using the sea surface microlayer as a vector. Trapping in the intertidal zone is believed to play an important role in this estuarine filter, and high-resolution sampling over two tidal cycles was used to investigate this trapping in a temperate salt marsh in Southampton Water, UK. Samples showed a significant decrease in microfibre abundance from the flood tide to the ebb tide, on a spring and a neap tidal cycle. This potential within-marsh sequestration has implications for the moderation of land to sea fluxes of microplastics by, as well as for exposure risk in, intertidal wetlands. A number of processes are likely to be involved in this trapping, including trapping by vegetation and, as indicated here, the direct settling or interaction of MPs with exposed sediment surfaces.

### Sampling and analytical methods

Hythe salt marsh, located in Southampton Water, U.K. (Fig. 1), was selected as the field site for this study (Grid reference: SU 43,137 07,336). The site is located within the Hythe to Calshot marshes Site of Special Scientific Interest, the most extensive area of salt marsh in Southampton Water. The marshes were the first location where *Spartina* hybrids were found, and to this date, retain a wide range of *Spartina* species and genetic material.

Southampton Water, due to frictional effects and a distorted tidal curve, has an extended high water period with a ‘double high tide’ generating a long slack water period at high water (which can last for 2 h, Fig. 2c). Coupled with tidal asymmetry effects which drive lateral suspended sediment transport from the main channel to the intertidal zone^43^, this may provide enhanced trapping potential for microplastics in local marsh systems. At the Hythe site, there is a large tidal creek which is accessible and sheltered from passing boat traffic and most (though not extreme) weather conditions and waves. This creek was used as the study sampling point. As the SML’s presence is affected by wave and wind activity^44^, a sheltered location was essential to enable an uninterrupted time series of samples.

Salt marsh creek sampling was chosen in order to assess if there were differences between the microplastics (in terms of number and size distribution) entering the marsh on the flood tide and leaving on the ebb tide, which would give an indication of whether vegetative (and other) trapping is occurring in the salt marsh system. A similar sample method using bulk water samples has been used to assess the direction of sediment transport in a marsh^45^, and while the method cannot be used to calculate an exact budget for the transport of sediment (or here, microplastics) into or out of the marsh it can give an indication of the direction of the overall transport, and (in our case) allow an assessment of trapping processes within the marsh system. Salt marsh flooding during tidal inundation occurs initially via creeks, followed by overtopping of the marsh surface. Draining during the ebb tide occurs in the opposite direction, with the marsh surface draining followed by the creeks. Consequently salt marsh creeks are the first and last element of a marsh to interact with the rising and falling tide, and provide a good indicator of temporal microplastic trends in the marsh system over a tidal cycle.

**Figure 6:**
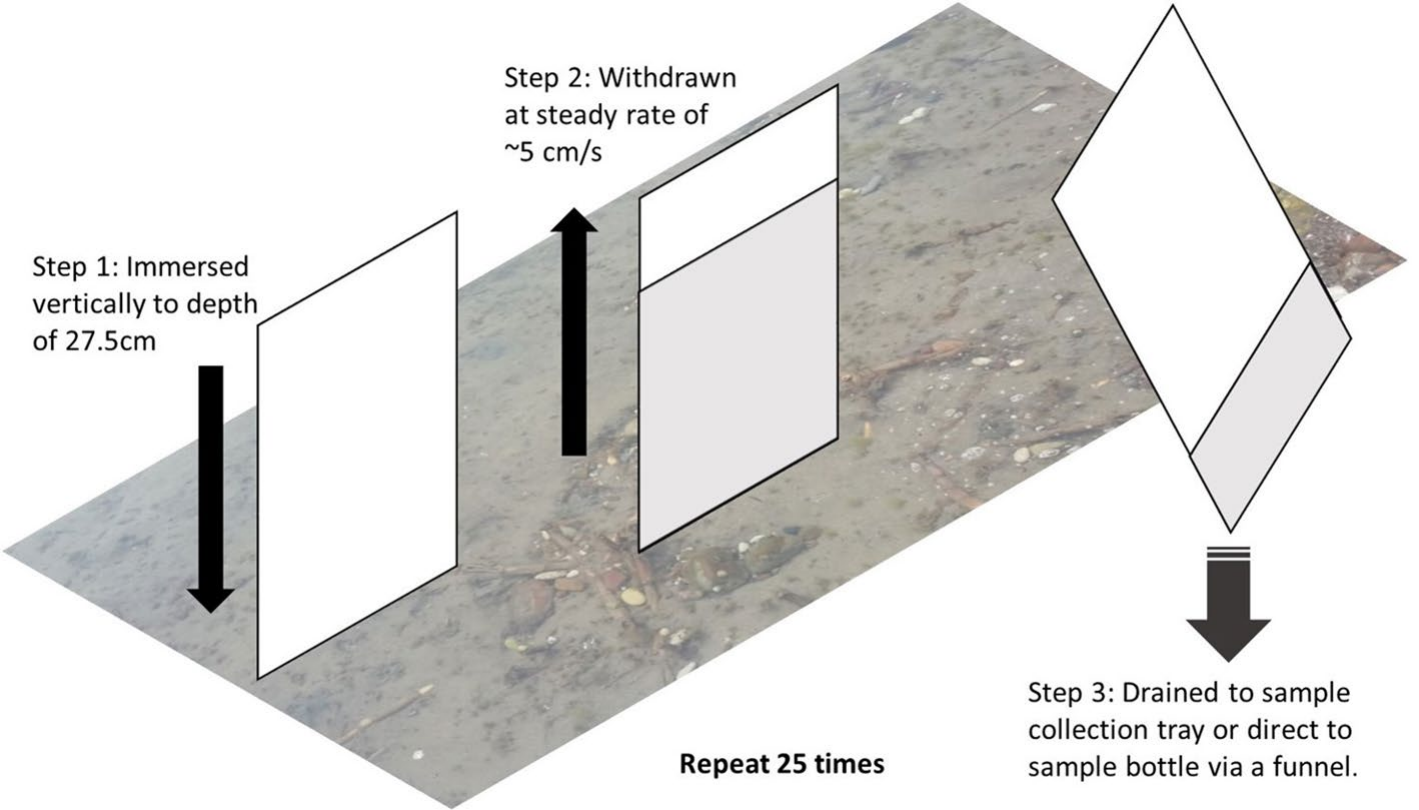
Schematic diagram of the glass plate sampling technique (after Anderson et al.^8^).

The method used for sampling microplastics in the SML was the glass plate method, previously trialled by Anderson et al*.*^8^. This method selectively samples the sea surface microlayer through surface tension, which removes the SML with the glass when the plate is removed from the water. Full details are given in Anderson et al*.*^8^*,* but in summary: a glass plate (18.2 × 30 × 0.4 cm) was immersed vertically into the water. This was initially carried out three times to rinse the glass plate with ambient water before use. The plate was then immersed to a set depth of 27.5 cm, withdrawn at a steady rate (~ 5 cm/s) and the draining water collected into a sample collection bottle (500 mL polyethylene terephthalate (PET)), pre-washed on site with local seawater (Fig. 6). This procedure was repeated for a total of 25 glass plate dips, collecting the subsequent sample water as a composite sample. Repeat sampling using this method, in similar sheltered locations, has shown good reproducibility between samples (< 3% variability, n = 3, authors’ unpublished data). Samples were taken every fifteen minutes on the flood and ebb tide, and once during the long high water period (*ca*. 2 h). Both spring and neap tides were sampled, on 13th September 2018 (spring tide, tidal range = 4.30 m, 13 samples total) and 5th October 2018 (neap tide, tidal range = 2.25 m, 9 samples total).

The sea surface microlayer samples were filtered onto 0.45 µm filters (cellulose nitrate, Whatman, Bucks., U.K.), without any pre-treatment due to their low sediment and organic matter content (samples showing slightly higher suspended sediment concentrations were filtered in two fractions, to reduce the amount of sediment on the filters for subsequent visual microplastic identification). To reduce the risk of sample contamination, which can be considerable^8,46,47^, several control measures were undertaken. These were as follows: wearing cotton clothing and cotton laboratory coats; working in a low-flow fume cupboard; running concurrent procedural blanks using MilliQ water; and leaving a dampened filter exposed as a laboratory blank. Contamination on these procedural and laboratory blank filters was low and consistent, with average contamination values subtracted from counts to give a lower bound for fibre abundance. Two procedural blanks consisting of MilliQ water were processed using the same techniques and at the same times as the sample filters, and a blank dampened filter was left exposed alongside the filtration equipment. The majority of fibres on these filters were transparent, so the results are corrected to remove all transparent fibres^8^ and corrected for an average of the coloured fibres found. This approach recognises the dominance of transparent fibres in control samples and is consistent with previously published work^8^.

Suspected microplastics were dominated by fibres (99.58%). To enumerate the fibres found, filters were examined in a row-wise fashion under an optical microscope (GX Microscopes, GXMXPL1530) at × 40 magnification. Filters were double-counted by one operator and counted again by a second. During one count, fibres were measured using a calibrated microscope camera (using a stage micrometre) and software (Dino-Eye, Dino-Lite Eyepiece Camera). Averages of these three counts, corrected for the fibres found on the blank filters, are presented here. Abundances were converted into fibres/m^2^ by calculating the area of glass plate used to sample, to enable comparability to previous work in the same estuary^8^. Volumetric abundances were calculated using the average depth of SML sampled (100 µm^8^) to enable additional comparison to other studies.

Identification of microplastics via visual processes has the potential to involve serious error and bias^48^. While it is the most common technique used to enumerate environmental microplastics^49^, it may lead to over- or under-estimation of plastic abundance^50^. However, considering the cost of analytical equipment^51^, and when used in conjunction with chemical analysis techniques, visual identification is an acceptable technique^52^. Published criteria for microplastic identification were followed when carrying out visual inspection of filters^53^.

In order to confirm microplastic identification, selected filters were subjected to Fourier Transform Infrared (FTIR) spectroscopic analysis. FTIR analysis is a common spectroscopic technique used to identify polymer chemistry of suspended microplastics, and can enable correction for misidentification of natural fibres. µATR-FTIR was undertaken with a Perkin Elmer Spotlight 400 Imaging system equipped with µATR accessory at the National Oceanography Centre (Southampton). Samples were re-filtered onto stainless steel filters, and (following observations of interference from sediment also on the filter), filters were treated using the Oil Extraction Protocol^54^ with the addition of soaking in Decon 90 to efficiently remove the oil. Thirty-two fibres were analysed using µATR-FTIR, or approximately 5% of the total fibres visually identified. The collected individual infrared spectra were exported into PerkinElmer Spectrum 10 software for identification. This involved the comparison of the measured spectra to the reference spectra in the polymer library (18,711 460 polymer types; spectra database from S.T. Japan-Europe GmbH, Germany/Japan). Spectra with the hit quality > 0.8 (score range 0 to 1) were accepted as verified polymers types. Note that in this case score of 0.8 or 80% corresponds to 80% similarity between the measured and the reference spectra.

Suspended sediment concentrations (SSC) were determined in bulk water samples taken simultaneously to the SML samples. These samples were taken by rinsing a 2 L PET bottle with ambient water, before opening and filling at approximately 5 cm water depth, shortly before the SML sample was taken, in the same location as the SML sample. A 50 mL aliquot was taken from the homogenised water sample, and filtered through a glass fibre (Whatman GF/C) filter before being dried at 60 °C overnight until constant weight.

When carrying out statistical analyses, a significance level of 95% was used throughout. Log-normal transformations were utilised to normalise fibre abundance, as tested by Shapiro–Wilk’s (*p* = 0.1033).

### Counting error and limit of detection estimation

Multiple counts of the fibres on the filters enabled the operator counting error to be calculated. This was calculated as 1 standard deviation as a percentage of the average count, per filter.

The limit of detection was calculated from the procedural and airborne blank samples collected during filtration. While clear fibres were discounted entirely from the analysis, coloured fibres were observed on the blank filters. The limit of detection used here (as shown in Fig. 2a,b) was calculated as the average of these observations + 3 standard deviations. This was then converted to number/m^2^ using the average area sampled. The limit of detection is indicated in Fig. 2 by the solid red horizontal line.

## Supplementary information

Supplementary Information.

## Data Availability

All relevant data are contained within the paper and the supplementary information, and are fully available without restriction.
