# Supplementary material for: Identification of tidal trapping of microplastics in a temperate salt marsh system using sea surface microlayer sampling
Source: Sci Rep. 2020 Aug 24;10:14147. doi: 10.1038/s41598-020-70306-5 (PMC7445233; doi:10.1038/s41598-020-70306-5)
Supplement: Supplementary file 1 — Supplementary Information. [file 41598_2020_70306_MOESM1_ESM.docx]

# **Identification of tidal trapping of microplastics in a temperate salt marsh system using sea surface microlayer sampling.**

*Jessica L. Stead^1*^, Andrew B. Cundy^1^, Malcolm D. Hudson^1^, Charlie E.L. Thompson^2^, Ian D. Williams^1^, Andrea E. Russell^1^ and Katsiaryna Pabortsava^3^*

** Corresponding author. Email: J.L.Stead@soton.ac.uk*

*^1^ University of Southampton, Southampton, SO14 3ZH*

*^2^ Channel Coastal Observatory, Southampton, SO14 3ZH*

*^3^ National Oceanography Centre, Southampton, SO14 3ZH*


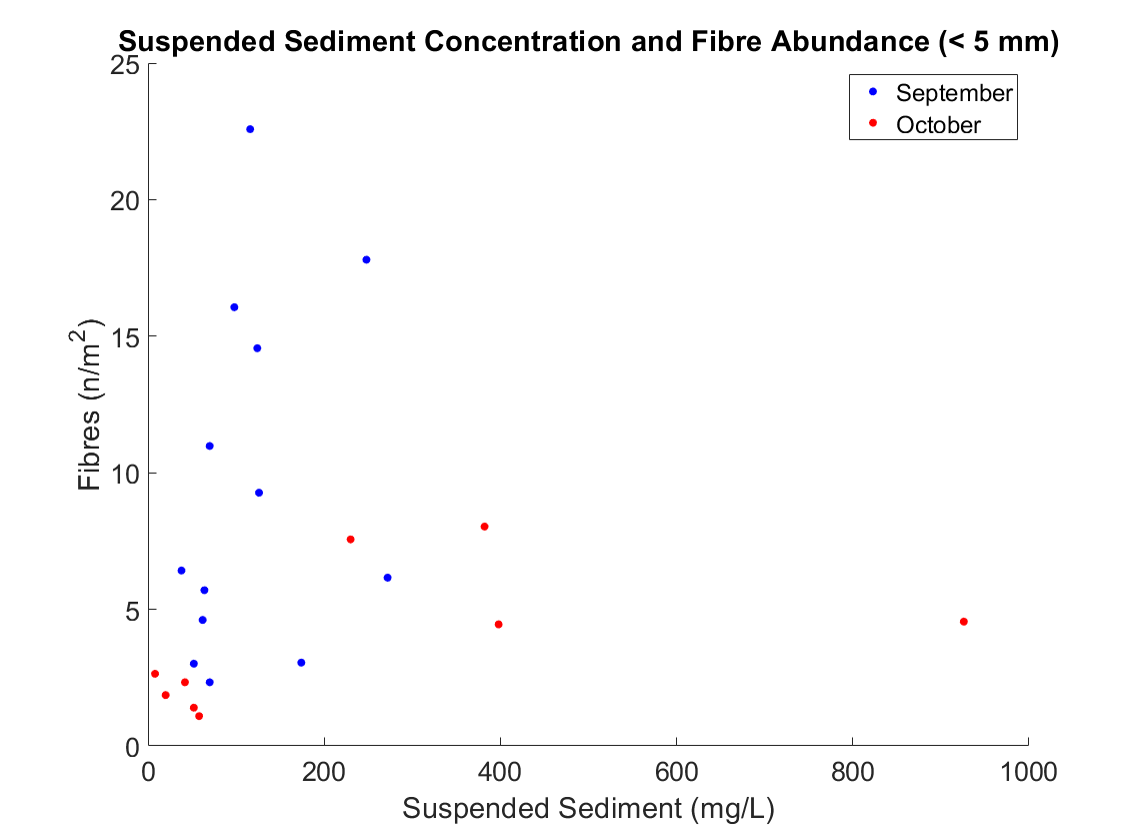


*Figure 1: Suspended sediment concentration obtained from the bulk water samples (5 cm depth); fibre abundance in the sea surface microlayer (SML) at the same time point.*
